# Vector Competence of *Aedes aegypti* from São Tomé and Príncipe for West Nile Virus Transmission

**DOI:** 10.3390/microorganisms12102038

**Published:** 2024-10-09

**Authors:** Rafael Marmé, Filipe Tomaz, Carla A. Sousa, João Pinto, Gregory C. Lanzaro, Ricardo Parreira, Gonçalo Seixas

**Affiliations:** 1 Global Health and Tropical Medicine (GHTM), Associate Laboratory in Translation and Innovation Towards Global Health, LA-REAL, Instituto de Higiene e Medicina Tropical (IHMT), Universidade Nova de Lisboa, UNL, Rua da Junqueira 100, 1349-008 Lisboa, Portugal; prmartinsm@gmail.com (R.M.); tomazfilipe88@gmail.com (F.T.); casousa@ihmt.unl.pt (C.A.S.); jpinto@ihmt.unl.pt (J.P.); ricardo@ihmt.unl.pt (R.P.); 2 Vector Genetics Laboratory, Department of Pathology, Microbiology and Immunology, University of California, 1089 Veterinary Medicine, 4225 V3 MB, Davis, CA 95616, USA; gclanzaro@ucdavis.edu

**Keywords:** *Aedes aegypti*, West Nile virus, vector competence, São Tomé and Príncipe, arbovirus transmission

## Abstract

The global distribution of *Aedes aegypti* mosquitoes, particularly in tropical regions, poses a significant public health risk due to their apparent ability to transmit arboviruses such as West Nile virus (WNV). This study aimed to evaluate the vector competence of *Ae. aegypti* from São Tomé and Príncipe (STP) for the transmission of the WNV PT6.39 strain, considering its potential role as a bridge vector in a region where *Culex quinquefasciatus* would be the main vector. *Aedes aegypti* mosquitoes were collected, reared, and experimentally infected with WNV, with viral dissemination and transmission potential assessed 7, 14, and 21 days post infection (dpi). The results showed an increasing trend in infection rates, from 5% at 7 dpi to 35% at 21 dpi, with corresponding dissemination rates of 0%, 100%, and 43%. The transmission rates also increased from 0% at 7 dpi to 67% at 21 dpi, with a maximum transmission efficiency of 10% observed at the final time point. Although *Ae. aegypti* from STP demonstrated the potential to transmit WNV, the overall transmission efficiency remained relatively low. These findings provide necessary insights into the vector competence of *Ae. aegypti* in this region, highlighting the importance of continued monitoring and targeted vector control measures to mitigate the risk of potential WNV outbreaks.

## 1. Introduction

The West Nile virus is an arthropod-borne virus transmitted by mosquitoes, particularly species within the *Culex* genus, and it has caused outbreaks in various regions around the world. The WNV exists in several lineages, with lineages 1 and 2 being the most commonly associated with human disease [1]. However, the role of other mosquito species, such as *Ae. aegypti*, in WNV transmission has gained increasing attention, particularly in regions where this species is prevalent. The global expansion of *Ae. aegypti*, particularly into new and previously unaffected regions, underscores the importance of understanding its vector competence for various viruses, including WNV [2].

São Tomé and Príncipe, a small island nation in the Gulf of Guinea, presents a unique ecological environment where *Ae. aegypti* thrives. Entomological studies in STP have documented the widespread presence of *Ae. aegypti* across the island, particularly in urban areas like Água Grande [3]. The potential for WNV transmission in this context is concerning, given that both *Ae. aegypti* and *Ae. albopictus* are established vectors in the region, even though WNV has not been documented. The 2022 dengue outbreak in São Tomé highlighted the high risk posed by these vectors, with *Ae. aegypti* exhibiting resistance to certain insecticides, complicating control efforts [3]. Despite the recognized presence of *Ae. aegypti* and its established role in transmitting other viruses, there has been limited research on its potential to transmit WNV in this region [4]. *Culex* species can serve as efficient enzootic or amplifying vectors for WNV. While several *Aedes* and *Ochlerotatus* species are highly efficient vectors under laboratory conditions, their feeding preferences suggest that they are more likely to act as bridge vectors between the avian *Culex* cycle and mammalian hosts rather than maintaining WNV in nature [5,6]. Given that *Culex quinquefasciatus* would likely be the primary vector of WNV in STP, studying the vector competence of *Ae. aegypti* is key to understanding its potential role as a bridge vector in the transmission cycle of WNV in this region. Previous studies have demonstrated that *Ae. aegypti* can transmit WNV under laboratory conditions, although typically less efficient than *Culex* mosquitoes [4]. This knowledge could inform public health strategies and vector control efforts, particularly in the context of emerging and re-emerging arboviral threats [7,8].

The factors influencing vector competence, including genetic variation, environmental conditions, and viral strain differences, are complex and not fully understood [9]. Moreover, vector competence can vary significantly based on geographic location, highlighting the need for region-specific studies [10,11]. This is particularly important in regions like STP, where ecological conditions may differ from those in regions where *Ae. aegypti* has been more extensively studied.

The present study aims to assess the vector competence of *Ae. aegypti* from STP for WNV transmission, using the PT6.39 strain of the virus [12]. By evaluating the infection, dissemination, and transmission rates at different time points post infection, this study aims to provide a comprehensive understanding of the potential role of *Ae. aegypti* in WNV transmission in this region. The findings of this study could have significant implications for public health, particularly in guiding vector control measures and monitoring efforts aimed at mitigating the risk of WNV outbreaks in STP.

## 2. Materials and Methods

### 2.1. Cell Culture

African green monkey kidney cells (Vero E6) were employed for the production and quantification of WNV due to their high susceptibility to viral infections and robust performance in virological assays. These cells were cultured in vented T25 or T75 cell culture flasks using Dulbecco’s Modified Eagle Medium (DMEM) (Gibco, Thermo Fisher Scientific, Waltham, MA, USA), supplemented with 10% Fetal Bovine Serum (FBS, Sigma-Aldrich, St. Louis, MO, USA) and 1% Penicillin–Streptomycin (Pen/Strep, Sigma-Aldrich, St. Louis, MO, USA) to ensure optimal growth and viability.

Vero E6 cells were maintained under standard cell culture conditions in a humidified incubator (Heraeus Heracell 240 CO_2_ Incubator, Thermo Fisher Scientific, Waltham, MA, USA) at 37 °C with 5% CO_2_. Subculturing was performed when the cells reached near-confluency to prevent overgrowth. The growth medium was removed, and the cells were washed with Dulbecco’s Phosphate-Buffered Saline (DPBS, Gibco) to remove the residual serum. A 1× Trypsin–EDTA solution (Gibco) was then added to detach the cells, followed by incubation at 37 °C for 3 min. After detachment, the trypsin was neutralized with DMEM containing 5% FBS, and the cells were reseeded at the desired density into new flasks containing fresh, pre-warmed medium.

### 2.2. Virus Production and Quantification

The WNV strain PT6.39 (GenBank accession number AJ965630.2) was used in this study. The virus was propagated from a stock with a concentration of 3.6 × 10^7^ plaque-forming units (PFUs)/mL. Vero E6 cells at approximately 90% confluency were infected in T75 flasks with a multiplicity of infection (MOI) of 0.1. The virus was allowed to adsorb into the cells for one hour, after which the inoculum was removed, and fresh medium was added. The flasks were then incubated at 37 °C with 5% CO_2_ for three days. Following incubation, the supernatant containing the virus was collected, centrifuged at 3000× *g* for 5 min to remove cellular debris, and stored in cryogenic vials (Corning Incorporated, Corning, NY, USA) at −80 °C for future use.

Viral titration was performed using a plaque assay on Vero E6 cells. The cells were seeded into 12-well plates (Corning Incorporated, Corning, NY, USA) and incubated overnight at 37 °C with 5% CO_2_ to achieve approximately 90% confluency. Serial tenfold dilutions of the stored virus were prepared, and 400 µL of each dilution was added to the respective wells. The plates were incubated for one hour, with gentle rocking every 20 min. Following incubation, 1 mL of overlay medium, consisting of a 1:1 mixture of 2× DMEM with 2% FBS and 2% Pen/Strep with 1.5% high-viscosity carboxymethyl cellulose (CMC), was added to each well. The plates were incubated at 37 °C with 5% CO_2_ for three days. After incubation, the cells were fixed with 3.7% formaldehyde, stained with crystal violet, and the plaques were counted to determine the viral titer, expressed as PFU/mL. The virus produced and quantified was 2 × 10^7^ PFU/mL and was used for the subsequent experiments.

### 2.3. Mosquito Collection and Rearing

*Aedes aegypti* eggs were collected using ovitraps placed in the Água Grande district in São Tomé during 2023 with all the necessary permits and approvals obtained from the local authorities for the collection and exportation of biological material. The eggs were transported to the “In Vivo Arthropod Security Facility” (VIASEF, IHMT-NOVA, Lisbon, Portugal) and reared under controlled conditions (27 ± 1 °C, 60–70% relative humidity, 12:12 light–dark cycle). Adult mosquitoes were fed a 10% sucrose solution and provided blood meals two to three times per week to facilitate egg production. These conditions were maintained to ensure the health and reproductive viability of the mosquito colony for subsequent experiments. The F5 generation was used for the experimental infections.

### 2.4. Mosquito Infection

Approximately 100 five-day-old *Ae. aegypti* mosquitoes were selected for experimental infections. The mosquitoes were housed in an Arthropod Containment Level 3 (ACL3) laboratory at VIASEF under standard conditions (27 °C, 70% relative humidity, 12:12 light–dark cycle). The sugar source was removed 24 h before being offered a blood meal (e.g., defibrinated sheep blood) mixed with WNV in a 1:1 ratio (final titer of 1 × 10^7^ PFU/mL) using the Hemotek feeding system (Hemotek Ltd., Great Harwood, UK). This viral dose, previously used to infect *Cx. quinquefasciatus* at VIASEF, has been associated with high rates of infection, dissemination, and transmission. The mosquitoes were allowed to feed for 30 min. Engorged females were anesthetized using CO_2_, separated, and maintained in groups of 25, with a 10% sucrose solution provided ad libitum. At 7, 14, and 21 days post infection (dpi), 20 mosquitoes were dissected, and their heads, bodies, and saliva were collected for subsequent analysis. Briefly, the wings and legs were removed to induce salivation, and the mosquitoes were placed in micropipette tips containing 10 µL of FBS for 30 min. Following the salivation period, mosquitoes were dissected into the abdomen (body) and thorax/head, which were placed into separate 2 mL tubes with 300 µL of 1× DMEM supplemented with 10% FBS and 1% Pen/Strep. The micropipette tips containing saliva were emptied into 0.2 mL centrifuge tubes pre-filled with 90 µL of 1× DMEM supplemented with 1% Pen/Strep. All samples were promptly stored at −80 °C.

The mosquito heads and bodies were homogenized using a Precellys Evolution Homogenizer (Bertin Technologies, Montigny-le-Bretonneux, France) with five 2 mm glass beads per tube. Samples were processed in two cycles at 2000 rpm for 20 s each, followed by centrifugation at 5000× *g* for 5 min to clarify the supernatant.

### 2.5. RNA Isolation and qRT-PCR

RNA was extracted using the NZYol reagent (NZYtech, Lisbon, Portugal), following the manufacturer’s protocol. Homogenized mosquito tissues (100 µL) and saliva (50 µL) were mixed with NZYol, followed by chloroform for phase separation. RNA was precipitated with isopropyl alcohol, washed with ethanol, and resuspended in RNase-free water. Samples were stored at −20 °C until further analysis.

WNV RNA was detected using a one-step RT-qPCR assay with primers and probes targeting the WNV genome [13]. RT-qPCR was performed in a 10 µL reaction volume, and viral RNA quantification was achieved using a standard curve generated from serial dilutions of WNV RNA.

### 2.6. Data Analysis

Infection rate (IR), dissemination rate (DR), transmission rate (TR), and transmission efficiency (TE) were calculated as follows:

- Infection rate (IR): proportion of mosquitoes with WNV-positive bodies out of the total number tested.
- Dissemination rate (DR): proportion of mosquitoes with WNV-positive heads + thoraxes out of the total number of infected mosquitoes.
- Transmission rate (TR): proportion of mosquitoes with WNV-positive saliva out of the total number of mosquitoes with disseminated infection.
- Transmission efficiency (TE): proportion mosquitoes with WNV-positive saliva out of the total number of mosquitoes tested.

A statistical analysis was performed using GraphPad Prism 8.0 (GraphPad Software, San Diego, CA, USA). Differences among the time points were assessed using the Kruskal–Wallis test, with a *p*-value of <0.05 being considered statistically significant.

## 3. Results

The population of *Ae. aegypti* from STP exhibited variable rates of infection, dissemination, transmission, and transmission efficiency when exposed to the WNV PT6.39 strain for 21 days post infection (dpi) (Table 1).

The infection rate increased over time, starting at 5% at 7 dpi, reaching 20% at 14 dpi, and 35% at 21 dpi. This indicates a gradual increase in the proportion of WNV-positive bodies among the exposed mosquitoes. The dissemination rate was observed at 0% at 7 dpi, significantly increasing to 100% at 14 dpi, and decreasing to 43% at 21 dpi. This suggests that the virus successfully disseminated within the mosquitoes by 14 dpi but did not maintain a consistent high rate by 21 dpi. The transmission rate was not observed at 7 dpi but increased to 25% at 14 dpi and further to 67% at 21 dpi, indicating that mosquitoes with disseminated infections were more likely to have virus-positive saliva as the infection progressed. The transmission efficiency followed a similar trend, being 0% at 7 dpi, 5% at 14 dpi, and reaching 10% at 21 dpi, calculated based on the total number of mosquitoes tested (N = 20). This shows that, while the proportion of mosquitoes capable of transmitting WNV increased over time, the overall efficiency remained relatively low.

**Figure 1:**
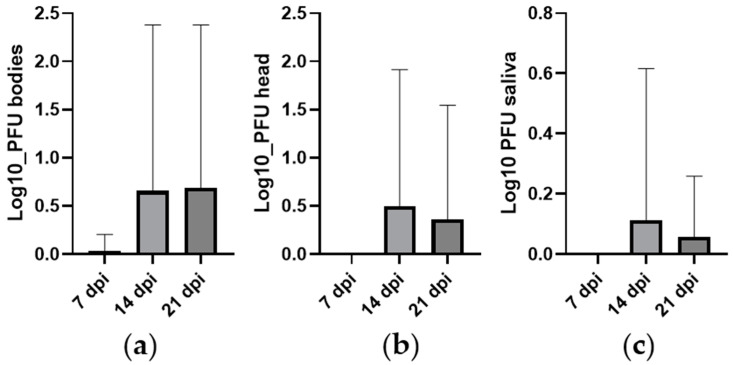
Number of viral particles in the body (**a**), head/thorax (**b**), and saliva (**c**) of *Ae. aegypti* from STP exposed to WNV at 7, 14, and 21 dpi. Error bars represent the standard deviation. The *y*-axis scales vary between tissues.

The log10 viral titers in the body, head/thorax, and saliva of infected *Ae. aegypti* were measured at 7, 14, and 21 dpi (Figure 1). The mean viral titers were generally low across all tissues and time points, with the highest mean titer observed at 14 dpi. The standard deviation was relatively high at 14 and 21 dpi, indicating variability in viral replication among individual mosquitoes.

The mean viral titer in the bodies increased slightly over time, with a mean of 0.037 at 7 dpi, 0.656 at 14 dpi, and 0.689 at 21 dpi. However, the Kruskal–Wallis test showed no statistically significant difference in viral titers across the different time points (χ^2^ = 5.36, df = 2, *p* = 0.069). Similar to the bodies, viral titers in the heads/thorax remained low across all time points, with a mean of 0.492 at 14 dpi and 0.359 at 21 dpi. The Kruskal–Wallis test also showed no significant differences across time points (χ^2^ = 4.15, df = 2, *p* = 0.126). Viral titers in the saliva were mostly low, with a mean of 0.112 at 14 dpi and 0.056 at 21 dpi. The Kruskal–Wallis test indicated no significant differences across the time points (χ^2^ = 2.00, df = 2, *p* = 0.368).

Although viral titers varied slightly between different tissues and time points, no significant differences were detected overall. This suggests that, while some mosquitoes did develop higher viral loads, the overall replication and transmission potential of WNV in the *Ae. aegypti* population from STP remained relatively low.

## 4. Discussion

This study provides important insights into the vector competence of *Ae. aegypti* from STP for WNV transmission. Since WNV has not been documented in STP, this study represents a proactive approach to understanding the potential risk if the virus were to be introduced. Human-mediated activities, such as international travel and trade, could facilitate the accidental introduction of WNV into the island. Tourists or imported goods could inadvertently bring infected mosquitoes or animals into STP. Additionally, infected migratory birds traveling through WNV-endemic regions could serve as reservoirs and introduce the virus during their stopovers in STP. Conducting vector competence studies in regions where WNV is not currently present is crucial as these studies help predict and prepare for the potential introduction and spread of the virus.

Our results show that, while *Ae. aegypti* in this region can become infected with WNV, the overall transmission potential remains relatively low, as indicated by the infection, dissemination, and transmission rates and the transmission efficiency observed for 21 days post infection. The infection rate increased from 5% at 7 dpi to 35% at 21 dpi, suggesting that *Ae. aegypti* from STP are susceptible to WNV infection, though less than other mosquito species, such as *Culex*, which are typically considered more competent vectors for WNV [4]. This lower susceptibility may be attributed to genetic differences between mosquito populations, environmental factors, or the viral strain used in this study [2,14,15,16]. The dissemination rates, which peaked at 100% at 14 dpi and then declined to 43% at 21 dpi, indicate that, while the virus successfully spread within the mosquitoes, a sustained high dissemination was not achieved. This trend could reflect a decrease in viral replication efficiency over time, possibly due to immune responses within the mosquitoes or the depletion of viral resources. This pattern is consistent with findings from other studies, where *Ae. aegypti* exhibited variable dissemination and transmission capabilities for arboviruses [17]. The transmission rates and efficiency, which increased over time but remained low overall, further support the conclusion that *Ae. aegypti* from STP are less effective WNV vectors compared to other mosquito species (e.g., *Culex* sp.). The low transmission efficiency particularly suggests that, even when the virus disseminates among the mosquitos, only a small proportion of mosquitoes can effectively transmit the virus through their saliva. This could be due to barriers within the mosquitos that prevent the virus from reaching the salivary glands or a limitation in the virus’s ability to replicate in those glands [10,18]. The process of viral transfer from the mosquito midgut to its saliva is a critical step in arbovirus transmission. Recent research has focused on the role of the salivary gland barrier in determining the efficiency of virus transmission [19,20,21,22]. This barrier can limit viral entry into the salivary glands, and successful arbovirus transmission requires the virus to overcome this obstacle. Factors such as viral tropism, mosquito immune responses, and the integrity of the salivary gland barrier influence whether the virus is transmitted in mosquito saliva. The findings from our study, which demonstrated a low overall transmission efficiency, could be partly attributed to such barriers. Further research investigating the molecular mechanisms that govern virus entry into the salivary glands could clarify why only a small proportion of mosquitoes in our study were capable of transmitting WNV.

One important consideration in vector competence studies is the potential influence of insect-specific viruses (ISVs) that naturally infect mosquitoes but do not replicate in vertebrate hosts [23,24]. These viruses can modulate mosquito immune responses and have been shown to affect the replication and transmission of arboviruses, such as WNV. For instance, studies have demonstrated that co-infection with *Culex* flavivirus (CxFV), an ISV, can reduce WNV transmission efficiency in *Culex* mosquitoes by inhibiting the replication of WNV in co-infected mosquitoes [25]. Similarly, Goenaga et al. (2015) found that co-infection with Nhumirim virus (NHUV), another ISV, could block WNV transmission in *Culex* mosquitoes, likely through interference with the virus replication mechanisms [26]. Although our study did not screen for the presence of ISVs in the *Ae. aegypti* population from STP, potential co-infection with ISVs may have influenced the observed infection, dissemination, and transmission rates. Future research should include screening for ISVs to evaluate their role in modulating vector competence, as co-infection with ISVs could either enhance or reduce WNV transmission. Understanding these interactions could provide further insights into the epidemiological dynamics of WNV in STP and help refine vector control strategies.

Our findings align with previous research that has demonstrated the relatively low vector competence of *Ae. aegypti* for WNV under laboratory conditions [2,4]. Studies have shown that *Ae. aegypti* is a more competent vector for other arboviruses, such as dengue virus and Zika virus, compared to WNV [27]. The decline in dissemination and the moderate increase in transmission rates over time observed in our study are consistent with the results of other studies that have examined the temporal dynamics of WNV infection in mosquitoes [28].

Given the limited vector competence observed in this study, future research should explore the impact of different viral doses on transmission outcomes, as higher doses may overcome some of the barriers to effective transmission observed at lower doses [29]. Additionally, it is crucial to test other WNV lineages, particularly lineage 2, which is known to circulate in nearby regions and may exhibit different patterns of infectivity and transmission in *Ae. aegypti* [30,31]. A key consideration in this study was the use of Vero cells to propagate WNV. While Vero cells are widely used for their permissiveness to viral infections, recent studies have highlighted structural differences in viral RNA when grown in Vero cells compared to mosquito-derived C6/36 cells. Specifically, viruses exhibit different replication dynamics and structural features in Vero cells compared to C6/36 cells, particularly in the context of the Zika virus [32]. This suggests that the three-dimensional structure of viral RNA in Vero cells may differ significantly from that in mosquito cells, which could influence viral replication dynamics and immune evasion strategies. These structural differences underscore the limitations of relying solely on Vero cells for virus propagation, particularly in studies aimed at investigating virus–vector interactions. Future research should incorporate the use of mosquito-derived cell lines, such as C6/36 cells, to produce viral stocks that more accurately reflect the virus’s behavior in its natural vector host. This approach would improve the translatability of laboratory findings to real-world transmission dynamics.

Moreover, the use of animal models to study WNV and mosquito interactions represents another promising avenue for future research. A recent study by [33] demonstrated the utility of an AG129 mouse model for studying WNV in *Cx. quinquefasciatus*. Applying similar models to assess the interactions of WNV with mosquitoes present in STP could yield insights relevant to local transmission dynamics.

Furthermore, the main vector for WNV transmission in many regions, *Cx. quinquefasciatus*, should be assessed in STP to better understand its role in local WNV transmission dynamics. Given that *Culex* species are generally more competent vectors for WNV, their role in STP could be significant, and monitoring their vector competence is essential for comprehensive vector control strategies. Additionally, it is important to acknowledge that the relatively small sample sizes in this study may limit the robustness of the statistical results. This limitation should be considered when interpreting the findings, and future studies with larger sample sizes will be essential to strengthen the conclusions drawn.

In addition to *Ae. aegypti*, *Ae. albopictus* has been identified as a potential vector for WNV in various regions [34,35,36]. The presence of *Ae. albopictus* in STP could represent an additional risk factor for WNV transmission, particularly in areas where it co-exists with *Ae. aegypti*. Future studies should access the vector competence of *Ae. albopictus* for WNV in this region to provide a more comprehensive understanding of potential dynamics of arbovirus transmission.

Given the complex interaction between environmental factors, mosquito species, and viral strains, ongoing surveillance is essential. Vector control strategies should be informed by localized ecological data to effectively mitigate the risk of WNV transmission. This approach could involve the enhanced monitoring of mosquito populations, particularly during peak transmission seasons, and the implementation of control measures that are tailored to the specific species potentially responsible for future WNV transmission in STP. Given the apparent absence of WNV in STP, vector control strategies should focus on preventing the introduction of the virus. This could include monitoring and controlling mosquito populations that are known to be competent vectors of WNV (e.g., *Cx. quinquefasciatus*). Surveillance efforts should include monitoring for WNV in *Aedes* species, *Cx. quinquefasciatus*, and migratory birds, which could serve as reservoirs for the virus [37,38].

Future research should focus on understanding the underlying factors that contribute to the variability in vector competence observed in different mosquito populations. Studies examining the genetic basis of vector competence, the impact of environmental factors, and the interactions between different arboviruses within the same mosquito population could provide valuable insights [39]. Additionally, field studies that assess the actual transmission dynamics of WNV in natural mosquito populations in STP would complement laboratory findings and help refine public health strategies.

## 5. Conclusions

In conclusion, while *Ae. aegypti* from STP can become infected with WNV, their overall competence as vectors is limited. This study contributes to the growing body of literature on mosquito-borne arboviruses in Africa and highlights the importance of region-specific research to guide effective vector control measures. Understanding the nuances of vector competence in different mosquito species is critical for preventing the spread of WNV and other arboviruses in endemic regions.

## Figures and Tables

**Table 1:** Infection, dissemination, transmission rates, and transmission efficiency (in %) of *Aedes aegypti* from São Tomé and Príncipe exposed to WNV PT6.39 strain.

Days Post Infection				
	IR % (*n*)	DR % (*n*)	TR % (*n*)	TE % (*n*)
7	5 (20)	0 (1)	0 (0)	0 (20)
14	20 (20)	100 (4)	25 (4)	5 (20)
21	35 (20)	43 (7)	67 (3)	10 (20)

*n*: the numbers in parentheses represent the total number of mosquitoes tested.

## Data Availability

All data generated in this study are available within this article.
